# Gun-Shot Wounds and Other Injuries of Nerves

**Published:** 1865-02

**Authors:** 


GUN-SHOT WOUNDS AND OTHER INJURIES OF NERVES. By S. WEIR MITCHELL, M.D., GEORGE R. MOREHOUSE, M.D., and WILLIAM W. KEEN, M.D., Acting Assistant-Surgeons U.S.A., in charge of U.S.A. Wards for Diseases of the Nervous System, Turner's Lane Hospital, Philadelphia. Philadelphia: J. B. LIPPENCOTT & Co. 1864.

This is an unbound monograph of 164 pages; containing much matter of interest, especially in relation to certain pathological conditions of the nervous and muscular systems, and their treatment. For sale by S. C. GRIGGS & Co., 41 Lake St., Chicago.

